# Study of a Fluorescent System Based on the Naphthalene Derivative Fluorescent Probe Bound to Al^3+^

**DOI:** 10.3390/mi14040868

**Published:** 2023-04-17

**Authors:** Qiuping Li, Lei Ma, Jianyan Li, Lijuan Wang, Liansheng Yu, Yuehui Zhao, Yuguang Lv

**Affiliations:** 1 College of Pharmacy, Jiamusi University, Jiamusi 154007, China; 2 Inspection and Testing Center, Jiamusi 154007, China

**Keywords:** naphthalene derivatives, fluorescence probe F6, Al^3+^ detection

## Abstract

The naphthalene derivative fluorescent probe F6 was synthesized and a 1 × 10^−3^ mol/L solution of Al^3+^ and other metals to be tested was prepared for the subsequent experiments. The Al^3+^ fluorescence system of the naphthalene derivative fluorescent probe F6 was successfully constructed as demonstrated by fluorescence emission spectroscopy. The optimal time, temperature and pH of the reaction were investigated. The selectivity and anti-interference ability of the probe F6 for Al^3+^ were investigated by fluorescence spectroscopy in a methanol solution. The experiments showed that the probe has high selectivity and anti-interference ability for Al^3+^. The binding ratio of F6 to Al^3+^ was 2:1, and the binding constant was calculated to be 1.598 × 10^5^ M^−1^. The possible mechanism of the binding of the two was speculated. Different concentrations of Al^3+^ were added to Panax Quinquefolium and Paeoniae Radix Alba. The results showed that the recoveries of Al^3+^ were 99.75–100.56% and 98.67–99.67%, respectively. The detection limit was 8.73 × 10^−8^ mol/L. The experiments demonstrated that the formed fluorescence system can be successfully adapted for the determination of Al^3+^ content in two Chinese herbal medicines, which has good practical application.

## 1. Introduction

In daily life, people come into frequent contact with products containing Al^3+^, such as traditional Chinese medicine, western medicine, doughnuts, food packaging, knitted products, and automobiles. However, with the continuous development of scientific research, it has been found that excess Al can cause a series of metabolic disorders, accelerate aging and lead to brain cell damage [1]. Therefore, the development of a rapid and simple Al^3+^ detection technique has become a current research hotspot. There are various methods for the detection of metal ions, such as atomic spectrometry, electrochemical analysis, fluorescence spectrometry, UV-visible spectrometry, the biochemical method, the colorimetric method, etc., which have their own advantages and disadvantages. Fluorescence spectrometry has become the most common method for metal ion detection because of its high sensitivity, as well as its simplicity and rapidity compared with other methods [2,3].

A fluorescent probe can emit a fluorescent signal to a target to achieve analysis and detection of the target. Fluorescent probe detection technology uses fluorescent substances as detection indicators by irradiating them at specific wavelengths to excite them to a luminescent state or to undergo fluorescence bursts at specific wavelengths. The change in fluorescence emission intensity of the substance under test is used to quantitatively or qualitatively analyze the substance; therefore, fluorescent probes can use fluorescent signals to express the identification of ions, molecules and other substances [4,5]. In recent years, fluorescent probes based on various molecules such as rhodamine and naphthalene derivatives have been developed [6,7]. Naphthalene derivatives are a new class of fluorescent probes with a conjugated structure based on bisbenzyl rings. Also, naphthalene has good modifiability and can be modified by modifying its functional groups to achieve modulation of its optical properties [8,9]. As a novel fluorescent probe, naphthalene derivatives have excellent properties in fluorescence imaging, such as low cost, good stability, structural plasticity, good luminescence properties and high quantum efficiency [10,11].

The fluorescence probe responds quickly and can detect aluminum ions qualitatively and quantitatively through the linear relationship between fluorescence intensity and aluminum ion concentration, which provides a guarantee for real-time detection of aluminum ions. People are committed to constructing a fluorescent probe that can directly and rapidly detect Al^3+^. However, compared with other metal ions, Al^3+^ presents more difficulties in detection because of its poor coordination ability, strong hydration ability, and lack of spectral characteristics [12,13]. However, naphthalimide has the advantages of stable planar structure, large Stokes shift, high quantum yield, strong light resistance and low toxicity, so it is selected as a fluorescent group because of its excellent properties when constructing fluorescent probes for detecting Al^3+^ [14,15]. Naphthalimide derivatives have excellent photochemical and physical properties, which makes them become hot spots in fluorescence probes for detecting ions. Because of the “imide” group containing electron pulling action and the strong electron donor groups R, an electron transferred transition (ET) band is formed, producing a phenomenon of high fluorescence quantum yield. Researchers have designed different organic fluorescent probes by changing RR [16]. This probe has many advantages such as good selectivity, anti-interference, superior sensitivity, large Stokes shift and high fluorescence quantum yield, and has been widely used in chemistry, medicine and environmental science. With the development of the fluorescence detection industry, the mechanisms of action of fluorescent probes have become clear. The mechanisms of action of most fluorescent probes are mainly photo-induced electron transfer (PET), fluorescence resonance energy transfer (FRET), excited-state intramolecular proton transfer (ESIPT) and intramolecular charge transfer (ICT) [17,18]. In the past 20 years, people have improved their photophysical and photochemical properties by modifying the structure of naphthalimide. Various fluorescent probes are prepared by effectively combining the naphthalimide and the recognition group [19,20]. Vinita Bhardwaj’s team has an in-depth study of fluorescent chemosensors. In one study, they prepared Schiff base 2-hydroxynaphthalene hydrazide (L) and incubated it in DMSO/HEPES for AIE behavior. It was used to detect Al^3+^ and they explored the properties of this fluorescent sensor, including metal ion selectivity, reversibility and detection lines. Unusual aspects exist compared with previous studies. They performed quantum mechanical calculations in the gas phase using the DFT method to obtain the three-dimensional structure of the complex compound formed by the probe and Al^3+^, with the aim of exploring the mechanism of their binding [21]. Chemical sensors can be chosen intentionally depending on the purpose of the study. In the latest research, George A. Gamovt’s team has compiled a chemical sensor database based on different algorithms for the ability of organic compounds to sense metal ions. It contains information on a total of 965 suitable compounds. In addition, a Telegram bot was developed which allows for predicting the sensing ability of the novel chemical sensors basing on their SMILES (https://t.me/mol_sim_bot, accessed on 31 March 2023). It can help researchers not only to find suitable sensors but also to develop new ones. This has greatly contributed to the progress of research in fluorescent probes [22].

With the gradual entry of herbal preparations into the pharmaceutical market, the market demands for the quality of herbal medicines have been increasing, which requires a certain content of metal ions in herbal medicines. Among them, metal contamination is one of the main reasons for the deterioration of the quality of herbal medicines, and the metal content of leaf herbs is higher compared with other classes of herbal medicines [23,24]. The content of metals varies among the parts of herbal medicines, and lead, cadmium, mercury, copper, aluminum and iron are the more common metals in herbal medicines. Metal elements in herbal medicines mainly originate from chemical fertilizers, sewage, atmospheric pollution and the absorption of metals by plants themselves. The presence of excessive metal ions in herbal medicines can adversely affect the normal physiological functions of the organism, so it is necessary to develop methods for the detection of metal ions in herbal medicines.

In this paper, a naphthalene derivative-like fluorescent probe with Schiff base structure was designed and synthesized to detect Al^3+^ with high selectivity and sensitivity. The probe was first characterized by infrared spectroscopy, nuclear magnetic resonance and mass spectrometry to determine the structure of the compound molecules. Then fluorescence titration was performed to investigate the effects of different reaction times and reaction temperatures on the fluorescence intensity of the fluorescent system, and the detection limit, binding rate and reaction mechanism were investigated. Finally, the probe was applied to the detection of Al^3+^ content in Chinese herbal medicines.

## 2. Materials and Methods

### 2.1. Reagents and Instruments

Naphthol (AR), hexamethylene tetramine (AR) and NH_2_NH_2_·H_2_O (AR) were purchased from Sinopharm Chemical Reagent Co., Ltd., Shanghai, China; acetic acid (AR) was purchased from Shanghai Tree of Science & Technology Co., Ltd., Shanghai, China; absolute ethyl alcohol (AR) and metal salt solutions were purchased from Tianjin kaitong chemical reagent Co., Ltd., Tianjin, China.

FT-IR spectrometer (NEXUS-670), Nicolay corporation, Phoenix, AZ, USA; Nuclear magnetic resonance (AV-400) spectrometer, Bruker, Karlsruhe, Germany; 970CRT fluorescence spectrophotometer, Shanghai Instrument Analysis Factory, Shanghai, China; vacuum drying oven (DZF-6020A), Shanghai Kuntian Company, Shanghai, China; electric thermostatic water bath (HWS-12), Shanghai Yiheng Company, Shanghai, China.

### 2.2. Synthesis of Fluorescent Probe F6

Intermediate compound synthesis: In a three-necked round-bottom flask with a thermometer attached, 26.64 g of naphthol and 28.56 g of hexamethylenetetramine were combined to make 36 mL of acetic acid, 26.64 g of naphthol, with 20 min of refluxing while stirring. The flask was then slowly filled with 39.6 mL of concentrated sulfuric acid within 1 h and heated at 95–98 °C for 2 h while being stirred. The combined solution was chilled to 25 °C following the reaction. 120 mL of deionized water was added to a 1000 mL beaker equipped with a thermometer, a magnetic stirrer and an ice water bath. The mixture was carefully and gradually poured into the beaker while being continually swirled to maintain a constant temperature of 25 °C. Then, 240 mL of deionized water was added to the beaker after the combined solution had been introduced, and the beaker was then agitated at 20 °C for 1.5 h. After stirring, the beaker was left to stand for 12 h before being filtered. The filter cake was repeatedly rinsed in deionized water until the pH of the resulting solution was about 7. The washing was halted, and a yellow-brown solid compound was produced by drying it at 45 °C in a vacuum drying oven. The resulting chemical was known as 3 [25,26].

**Scheme 1:**
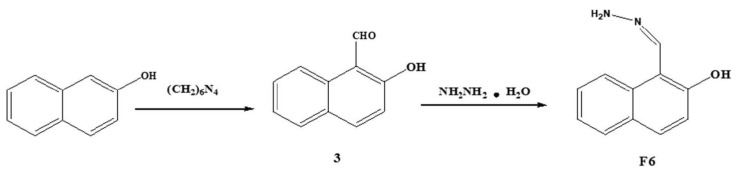
The synthetic route of the F6 probe.

Making a fluorescent probe: A quantity of 3 g of compound 3 was combined with 20 mL of NH_2_NH_2·_H_2_O and 100 mL of ethanol, and heated with reflux for 6 h. Then cooled to 25 ℃. To create the fluorescent probe, the combined solution was evaporated under decreased pressure, cleaned with ethanol and dried in a vacuum drying oven for 24 h (bright yellow powder). The F6 probe was used. Scheme 1 depicts the fluorescent probe F6’s synthesis process. [27].

### 2.3. Preparation of the Probe Solution and Metal Ion Solution

Weighed 3.72 mg of F6 probe was dissolved in CH_3_OH solution [28], and 2 × 10^−3^ mol/L probe solution was obtained in a 10 mL volumetric bottle. The 1 mL probe reserve solution was transferred into a 200 mL volumetric bottle, and the volume was fixed with CH_3_OH solution to obtain the probe solution (1 × 10^−5^ mol/L). This probe solution was used for subsequent measurements.

Weighed 133.34 mg of anhydrous aluminum chloride was dissolved in a small amount of deionized water and then shaken well with a constant volume of distilled water in a 10 mL volumetric flask to prepare a Al^3+^ stock solution (0.1 mol/L), which was stored in a cool and light-proof place. A quantity of 1 mL of the reserve solution was put into a 100 mL volumetric flask, and 1 × 10^−3^ mol/L of Al^3+^ solution was prepared with a constant volume of deionized water. Solutions of other metal ions (Cu^2+^, Co^2+^, K^+^, Mg^2+^, Mn^2+^, Pb^2+^, Zn^2+^, Fe^2+^, Na^+^, Ca^2+^, Ni^2+^, Cd^2+^, Fe^3+^, Sn^2+^, Hg^2+^) with the same concentrations were prepared by the above method.

### 2.4. Fluorescence System Determination

An appropriate amount of Al^3+^ solution was added to the probe solution. After a while, its fluorescence emission spectrum was scanned (excitation wavelength of 343 nm, sensitivity of 2). The fluorescence emission spectra of the pure probe solution were scanned by the same method.

### 2.5. Applied Research

Appropriate amounts of Panax Quinquefolium and Paeoniae Radix Alba were dried and ground separately, after which 0.6 g was weighed in a round-bottom flask, and appropriate amounts of Al^3+^ and mixed acid solutions (0.4 mL sulfuric acid, 0.6 mL perchloric acid, 6 mL nitric acid) were added for the spiked recovery experiment. The round-bottom flask was heated for a period of time to drive out the acid until the remaining volume of liquid did not exceed 1 mL, and deionized water was added to 50 mL (pH = 6). They were assayed separately for Al^3+^ content using the F6 probe. The recoveries and relative standard deviations were calculated. Four sets of experiments were performed for each sample [29,30].

## 3. Results and Discussion

### 3.1. Characterization of the Fluorescent Probe F6

**Figure 1:**
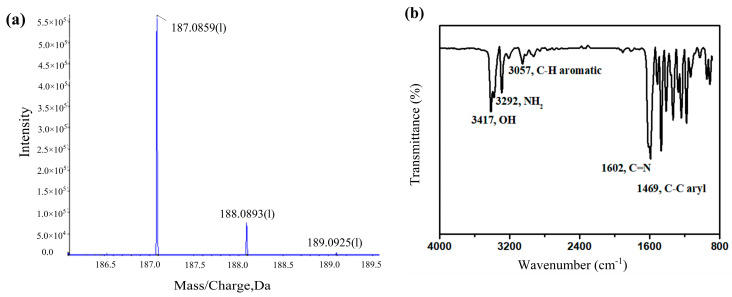
Mass spectrometry (**a**) and infrared spectroscopy (**b**) of the fluorescent probe F6.

To demonstrate the structure of the fluorescent probe F6, mass spectrometry and infrared spectroscopy were performed, and the results are shown in Figure 1. Figure 1a shows the mass spectrum of F6, from which it can be seen that the measured value of [M + H]^+^ was 187.0859, which was consistent with the expected relative molecular mass. Figure 1b shows the infrared spectrum of F6. From the figure, it can be seen that the peak of the probe molecule at 3057 cm^−1^ was the C-H stretching vibration peak, the peak at 1602 cm^−1^ was the C=N stretching vibration peak, the peak at 3417 cm^−1^ was the −OH stretching vibration peak, the peak at 3292 cm^−1^ was the N-H stretching vibration peak, and the peak at 1469 cm^−1^ was the backbone stretching vibration peak of the aromatic ring.

**Figure 2:**
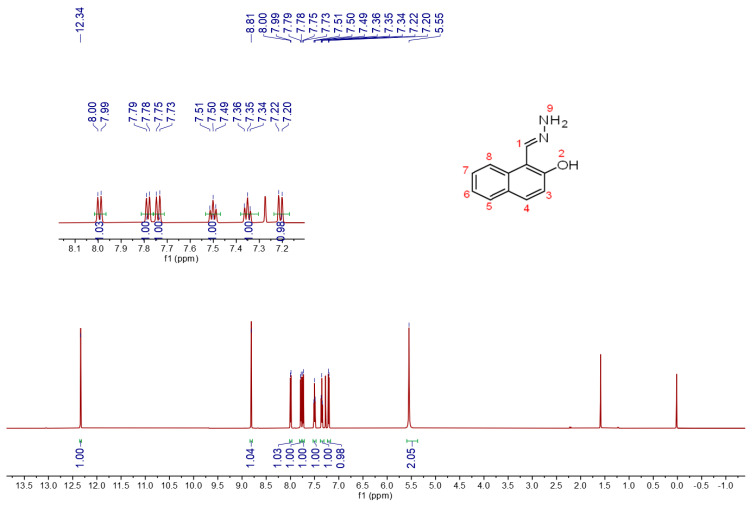
^1^H NMR spectra of the fluorescent probe F6.

Figure 2 shows the NMR hydrogen spectrum of probe F6. ^1^H NMR (600 MHz, CDCl_3_) δ: 12.34 (s, 1 H), 8.81 (s, 1 H), 7.99 (d, J = 8.6 Hz, 1 H), 7.78 (d, J = 8.1 Hz, 1 H), 7.74 (d, J = 8.9 Hz, 1 H), 7.50 (t, J = 7.7 Hz, 1 H), 7.35 (t, J = 7.4 Hz, 1 H), 7.21 (d, J = 8.9 Hz, 1 H), 5.55 (s, 2 H). The NMR carbon spectroscopy of probe F6. ^13^C NMR (151 MHz, CDCl_3_) δ: 157.61, 143.74, 131.46, 131.27, 129.05, 128.15, 127.04, 123.15, 119.90, 119.07, 108.63.

The analysis was carried out according to the hydrogen labeling above the structural. First, the H on the hydroxyl and imine were single peaks, 12.34 (s, 1 H) at 2 and 8.81 (s, 1 H) at 1, respectively; the amino-hydrogen 5.55 (s, 2 H) was at 9; the second region was the aromatic region, whose H chemical shifts were close together. The smallest H chemical shift at 3 was 7.21 (d, J = 8.9 Hz, 1 H). The adjacent four sites had coupling cleavage, so the coupling constant was consistent with 7.74 (d, J = 8.9 Hz, 1 H) for hydrogen at 4; the larger chemical shift was for H at 8, which cleaves into a double peak of 7.99 (d, J = 8.6 Hz, 1 H), while the same double peak at 5 was 7.78 (d, J = 8.1 Hz, 1 H); the remaining 7.50 (t, J = 7.7 Hz, 1 H), 7.35 (t, J = 7.4 Hz, 1 H) was for the H on 6 and H on 7. Finally, the hydrogen at 7.26 was the solvent peak and the one near 1.56 was the water-in-solvent peak. The NMR carbon spectrum showed that the synthesized probe F6 was equal to the expected number of carbons.

### 3.2. Fluorescence Spectrum of F6 Probe and Al^3+^

**Figure 3:**
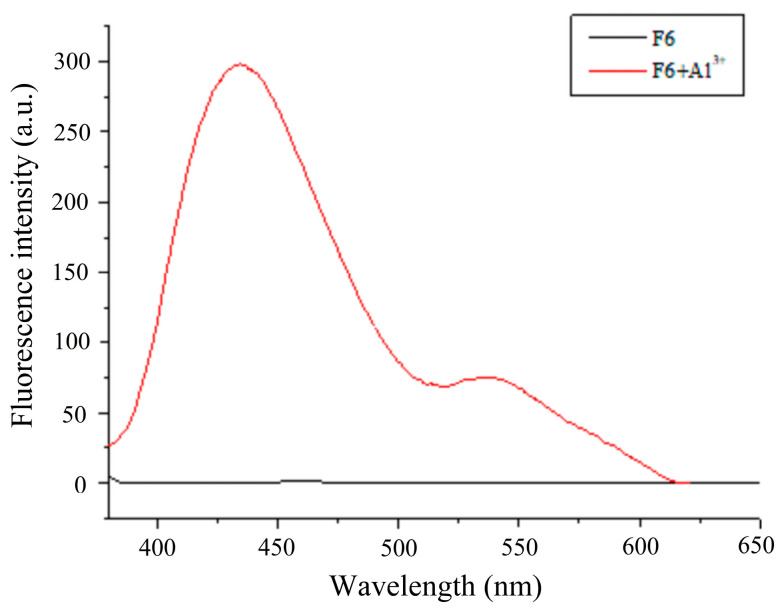
Fluorescence spectrum of F6 and Al^3+^ fluorescence systems.

Figure 3 shows the fluorescence emission spectra of the constructed Al^3+^ fluorescent system of the probe F6. There was a faint fluorescence peak at 462 nm when only pure probe solution was present. After a large amount of Al^3+^ was added to this probe solution, the fluorescence peak of the probe itself at 462 nm was blue-shifted and the fluorescence intensity was significantly enhanced, and a new fluorescence peak was formed at 538 nm. This newly appeared fluorescence peak was due to the binding of the probe F6 to Al^3+^. Thus, it can be inferred that the Al^3+^ fluorescence system of the F6 probe was successfully constructed.

### 3.3. Probe F6 Versus Al^3+^ Concentration and Standard Curve Plotting

**Figure 4:**
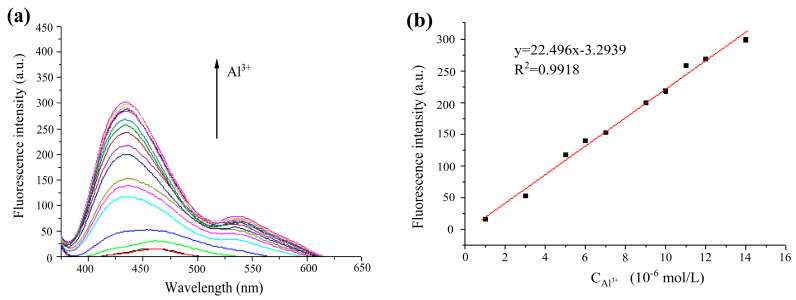
(**a**) Fluorescence spectra of F6 combined with Al^3+^ at different concentrations; (**b**) standard curve for the detection of Al^3+^ by fluorescence probe F6.

Figure 4a shows the fluorescence spectra of the fluorescent probe F6 (1 × 10^−5^ mol/L) bound to different concentrations of Al^3+^ (0–1.4 equivalents). It can be seen from the figure that the highest peak initially appearing at 462 nm shifted to 434 nm sequentially with increasing Al^3+^ concentration and its value also increased sequentially. Another new fluorescence peak gradually formed at 538 nm. When Al^3+^ reached 1.4 equivalents, the peak at 434 nm was basically unchanged, indicating that the binding of the probe to Al^3+^ reached saturation.

Figure 4b shows the standard curve for the detection of Al^3+^ by fluorescence probe F6. The results showed that the fluorescence intensity of the system was positively correlated with the content of Al^3+^. According to the standard curve, the fluorescence intensity of the system showed a good linear relationship with the concentration of Al^3+^, and the regression curve equation was y = 22.496x − 3.2939, R^2^ = 0.9918, with a linear range of 1 × 10^−6^–14 × 10^−6^ mol/L.

### 3.4. Study on Metal Ion Selectivity and Anti-Interference of Fluorescent Probe F6

**Figure 5:**
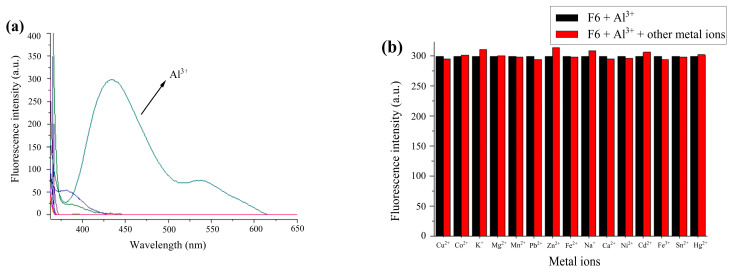
(**a**) Metal ion selectivity study; (**b**) Anti-interference study of fluorescent systems.

Figure 5a shows the fluorescence emission spectra of the F6 probe mixed with different metal ions. In order to better investigate the selectivity of F6 probe solution, Cu^2+^, Co^2+^, K^+^, Mg^2+^, Mn^2+^, Pb^2+^, Zn^2+^, Fe^2+^, Na^+^, Ca^2+^, Ni^2+^, Cd^2+^, Fe^3+^, Sn^2+^, Hg^2+^ and Al^3+^ solutions were added to the F6 probe solution. Compared with Al^3+^, when other metal ions were added, the fluorescence emission spectrum of the system did not change significantly, and the fluorescence intensity value was basically 0. Therefore, the F6 probe has good selectivity for Al^3+^.

Figure 5b shows the experimental results of the anti-interference ability of fluorescent probe F6 to recognize Al^3+^. In order to investigate the influence of other metal ions on the fluorescence system, the anti-interference experiment of the Al^3+^ fluorescence system was carried out. When 1.4 equivalents of Al^3+^ solution was added to the probe solution together with other metal ions, the probe still had a good ability to recognize Al^3+^. This indicates that the fluorescent system formed by the two has strong anti-interference ability.

### 3.5. Effect of Temperature and Reaction Time on Fluorescence System

**Figure 6:**
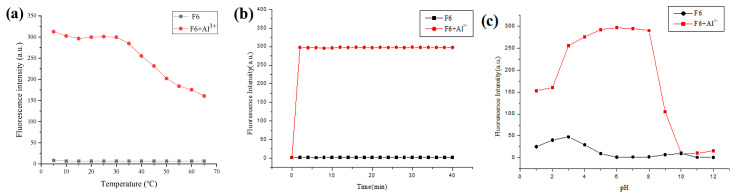
Effect of reaction temperature (**a**), reaction time (**b**), different pH (**c**) on the fluorescence system.

Figure 6a shows the influence of reaction temperature on fluorescence intensity when probe F6 detects an Al^3+^ fluorescence system. The influence of reaction temperature on fluorescence intensity was investigated in the range of 5~65 ℃. The results showed that the fluorescence intensity of the pure probe solution was not affected by temperature. When combined with Al^3+^, the fluorescence intensity of this system decreased with increasing temperature and remained stable in the range of 20~30 ℃. Therefore, we picked 25 °C as the optimal temperature for the reaction.

Figure 6b shows the influence of different reaction times on the fluorescence system. To determine the optimal time for the reaction of the fluorescent probe F6 and Al^3+^, the fluorescence intensity of the pure F6 solution and the system were measured 20 times in the range of 0 to 40 min. The results showed that the fluorescence intensity of the pure F6 solution was not affected by the reaction time. However, the fluorescence intensity of the system gradually increased with the increase in reaction time. after 2 min, the fluorescence intensity of the system was maximum and remained constant. For complete binding of F6 and Al^3+^, we picked 5 min as the optimal reaction time.

Figure 6c shows the effect of different pH (1–12) on the fluorescence intensity of the system when the F6 probe detects Al^3+^. The pH of the solution was configured by buffer solution. As shown in the figure, the fluorescence intensity of the pure probe increased with increasing pH in the range of 1–3, which may be due to the reaction between the hydrogen ions in the solution and the nitrogen atoms on the probe in the acidic environment. Additionally, the fluorescence intensity of the pure probe was stable at a pH of 6–8. In addition, the fluorescence intensity of the Al^3+^ system gradually increased and stabilized when the pH was 1–6. The maximum value was reached at pH = 6. When the pH value was greater than 6, the fluorescence intensity decreased to different degrees. It was speculated that the precipitation of Al(OH)_3_ was formed under alkaline environment, which led to the decrease of Al^3+^ concentration in the fluorescent system. After comprehensive analysis, the optimal pH condition for the reaction of this fluorescence system was determined to be 6.

### 3.6. Binding Ratio, Constants and Mechanism

**Figure 7:**
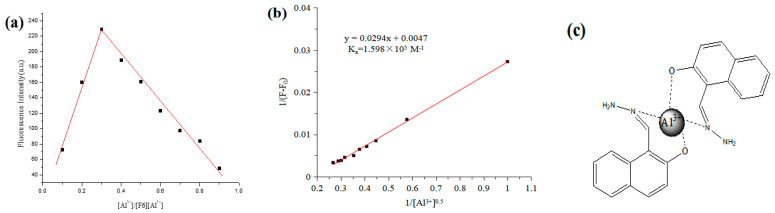
(**a**) The binding ratio of F6 to Al^3+^; (**b**) Benesi–Hildebrand plot of probe F6 with Al^3+^; (**c**) The possible binding mechanism of probe F6 to Al^3+^.

Figure 7a shows the binding ratio experiment of fluorescent probe F6 to Al^3+^. The concentration of Al^3+^ in the fluorescent system was varied and the fluorescence intensities were measured to determine the binding ratio of the probe F6 to Al^3+^. The vertical coordinate is the number of moles of Al^3+^, and the horizontal coordinate is the difference of fluorescence intensity. From the figure, it can be seen that the difference of fluorescence intensity was the largest when the molar ratio of Al^3+^ in the fluorescent system was 0.3. This suggests that the probe F6 bound to Al^3+^ in a 2:1 manner.

Figure 7b shows the Benesi–Hildebrand plot of the probe F6 with Al^3+^. From the fluorescence titration spectrogram, it is clear that the fluorescence intensity of the solution increases with increasing Al^3+^ content. The fluorescence intensity of the solution reached the maximum and remained constant when the Al^3+^ content was greater than 1.4 equivalents. The binding constants (*K_a_*) of the two can be calculated [31]:

$$\frac{1}{F-F_{0}}=\frac{1}{K_{a}F_{max}-F_{0}{\left[M^{X+}\right]}^{n}}+\frac{1}{F_{max}-F_{0}}$$

where *F* and *F*_0_ are the fluorescence intensity of probe F6 in the presence or absence of Al^3+^, respectively; *F_max_* is the fluorescence intensity of F6 when an excess of Al^3+^ is added; [*M^X+^*] is Al^3+^ concentration; *n* indicates the binding ratio of probe F6 to Al^3+^. The probe F6 binds to Al^3+^ in a 2:1 ratio, *n* = 0.5. The binding constant *K_a_* was calculated to be 1.598 × 10^5^ M^−1^, which indicated that the probe F6 had a strong binding capacity for Al^3+^.

Figure 7c shows the conjectured mechanism of the binding of the F6 probe to Al^3+^. As described in the references, the pure fluorescent probe solution almost did not fluoresce. The main reason for this phenomenon was the proton transfer and photoinduced electron transfer within the excited state molecule. The H on the phenolic hydroxyl group and the N on C = N underwent an excited state intramolecular proton transfer phenomenon, and the lone pair electron transfer of the N on C = N was a photoinduced electron transfer. The fluorescence intensity of the probe was enhanced by the addition of Al^3+^. This may be due to the fact that Al^3+^ inhibits the formation of hydrogen bonds between N and H and the lone pair electron transfer of N, thus leading to a decrease in intramolecular proton transfer and excited state photoinduced electron transfer. This phenomenon restored the fluorescence intensity of the F6 probe [15,26,32].

### 3.7. Determination of Detection Limit

**Figure 8:**
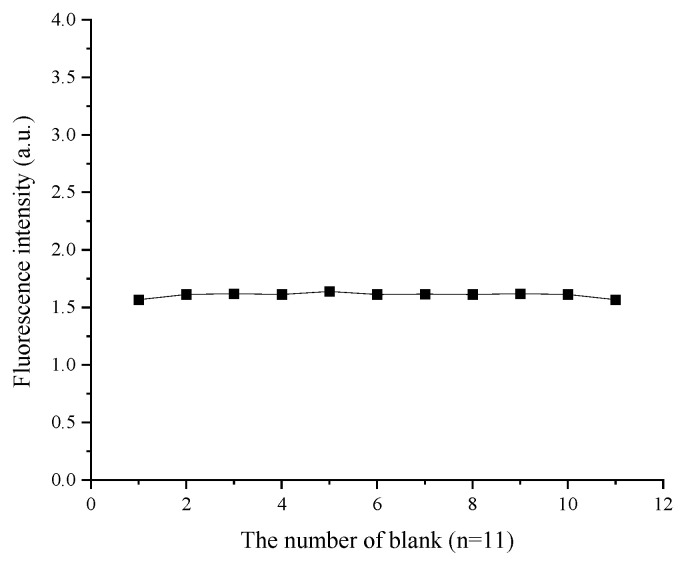
Fluorescence intensity measurement of pure probe solution (11 times).

Figure 8 shows a graph of the fluorescence intensity of the blank probe solution being measured 11 times. To investigate the detection limit of the fluorescent probe, the pure probe solution was measured in parallel and the fluorescence intensity was recorded. The standard deviation was calculated three times. The calculated value of δ was 0.655. Using the limit of detection equation: LOD = 3 δ/k, the limit of detection of Al^3+^ for the F6 probe was calculated to be 8.73 × 10^−8^ mol/L.

### 3.8. Methodological Examination

Table 1 shows the accuracy experiments of the constructed Al^3+^ fluorescence system. From the table, it can be seen that the RSD of the constructed Al^3+^ fluorescence system is 1.26%, and it was less than 2% under the same concentration. This indicates that the Al^3+^ fluorescence system has excellent precision.

Table 2 shows the accuracy results of the fluorescence systems formed by the F6 probe and Al^3+^. The recoveries of the three fluorescence systems measured at different Al^3+^ concentrations were 100.13 ± 0.96, 100.66 ± 0.86 and 98.98 ± 1.39, and the calculated RSD values were 0.96%, 0.85% and 1.4%, with RSD values less than 2%. This indicated that the detection accuracy of the constructed fluorescence system was good.

### 3.9. Reversibility of the Fluorescence System

**Figure 9:**
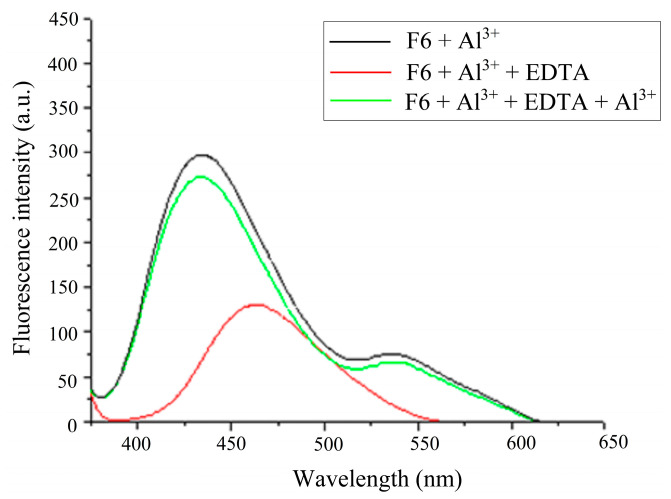
Reversibility study of fluorescent systems.

Figure 9 shows the reversibility of the Al^3+^ fluorescent system. In order to verify that the reaction between Al^3+^ and the probe molecule was reversible, EDTA was chosen to be added dropwise to the constructed Al^3+^ fluorescent system [33]. The fluorescence intensity of the fluorescence system was greatly reduced after the addition of EDTA solution. This is because EDTA is a complexing agent and possesses a strong complexing ability. When EDTA solution was added to this system, EDTA would form complexes with Al^3+^ and cause it to break away from the constructed probe fluorescence system. The fluorescence intensity was enhanced again when an excess of Al^3+^ solution was added to the mixed solution once again. In summary, the recognition of Al^3+^ by the F6 probe has some reversibility.

### 3.10. Applied Research

Table 3 shows the experiments for the determination of Al^3+^ content in Chinese herbal medicines. The content of Al^3+^ was determined by the blank spiking test method. The recoveries of Al^3+^ in Panax Quinquefolium and Radix Paeoniae Alba were in the ranges of 99.75–100.56% and 98.67–99.67%, respectively. The relative standard deviations were less than 2%. Therefore, this fluorescent probe can be used for the determination of Al^3+^ in Panax Quinquefolium and Radix Paeoniae Alba.

## 4. Conclusions

In this paper, the fluorescent system formed by the combination of naphthalene derivative fluorescent probe F6 and Al^3+^ was successfully constructed. Some investigations of this fluorescent system were carried out by fluorescence spectroscopy in methanol solution. The fluorescence system has a clear fluorescence intensity at 434 nm, and the fluorescence intensity increases with the increase of Al^3+^ concentration. It was demonstrated that the probe still has good selectivity for Al^3+^ in the presence of other metal ions. The fluorescence intensity of the pure probe solution was hardly affected by the reaction time and temperature; however, the Al^3+^ fluorescence system formed was somewhat affected. It is known from the experimental results that the optimal reaction temperature, time and pH were 25 °C, 5 min and 6, respectively. The fluorescent probe showed good linearity in the range of 1 × 10^−6^–14 × 10^−6^ mol/L with a detection limit of 8.73 × 10^−8^ mol/L. The binding ratio of the F6 probe to Al^3+^ was 2:1. a Benesi–Hildebrand plot was plotted and the binding constant *K_a_* = 1.598 × 10^5^ M^−1^ was calculated based on fluorescence titration experiments. The mechanism of their binding was explored. In particular, the probe has been successfully used to detect the Al^3+^ content in Panax Quinquefolium and Radix Paeoniae Alba, which has practical application.

## Figures and Tables

**Table 1:** Precision test of the F6 probe for Al^3+^ detection.

Number	ΔF	Average Value	RSD (%)
1	294.35		
2	293.18		
3	299.62		
4	299.85		
5	303.90	298.54	1.26
6	298.75		
7	293.97		
8	303.21		
9	300.98		
10	297.64		

**Table 2:** Accuracy test of the F6 probe for Al^3+^ detection.

[Al^3+^] (10^−6^ mol/L)	Recovery Rate (%)	Recovery Mean ± SD (%)	RSD (%)
3	99.81		
	101.21	100.13 ± 0.96	0.96
	99.37		
7	100.85		
	99.73	100.66 ± 0.86	0.85
	101.41		
11	97.45		
	99.31	98.98 ± 1.39	1.4
	100.17		

**Table 3:** Sample measurement results. (n = 8).

Sample	Addition Amount (1 × 10^−6^ mol/L)	Recovery Amount (1 × 10^−6^ mol/L)	Recovery Rate (%)	RSD(%) (n = 3)
Panax Quinquefolium	0	--	--	--
Panax Quinquefolium	5	4.99	99.8	0.87
Panax Quinquefolium	9	9.05	100.56	0.55
Panax Quinquefolium	12	11.97	99.75	0.63
Radix Paeoniae Alba	0	--	--	--
Radix Paeoniae Alba	5	4.96	99.2	0.94
Radix Paeoniae Alba	9	8.97	99.67	0.61
Radix Paeoniae Alba	12	11.84	98.67	1.02

## Data Availability

The data are unavailable due to privacy or ethical restrictions.
